# Antimicrobial Properties of Antidepressants and Antipsychotics—Possibilities and Implications

**DOI:** 10.3390/ph14090915

**Published:** 2021-09-10

**Authors:** Marina Caldara, Nelson Marmiroli

**Affiliations:** 1 Department of Chemistry, Life Sciences and Environmental Sustainability, University of Parma, Parco Area delle Scienze 11/A, 43124 Parma, Italy; nelson.marmiroli@unipr.it; 2 Interdepartmental Center SITEIA.PARMA, University of Parma, Parco Area delle Scienze 181/A, 43124 Parma, Italy; 3 Italian National Interuniversity Consortium for Environmental Sciences (CINSA), University of Parma, 43124 Parma, Italy

**Keywords:** antidepressants, antipsychotics, antimicrobials, drug repurposing

## Abstract

The spreading of antibiotic resistance is responsible annually for over 700,000 deaths worldwide, and the prevision is that this number will increase exponentially. The identification of new antimicrobial treatments is a challenge that requires scientists all over the world to collaborate. Developing new drugs is an extremely long and costly process, but it could be paralleled by drug repositioning. The latter aims at identifying new clinical targets of an “old” drug that has already been tested, approved, and even marketed. This approach is very intriguing as it could reduce costs and speed up approval timelines, since data from preclinical studies and on pharmacokinetics, pharmacodynamics, and toxicity are already available. Antidepressants and antipsychotics have been described to inhibit planktonic and sessile growth of different yeasts and bacteria. The main findings in the field are discussed in this critical review, along with the description of the possible microbial targets of these molecules. Considering their antimicrobial activity, the manuscript highlights important implications that the administration of antidepressants and antipsychotics may have on the gut microbiome.

## 1. Introduction

Life-threatening microbes are more and more often displaying insensitivity to the commonly used therapeutics, up to the point that antimicrobial resistance is nowadays considered a global challenge. Indeed, this phenomenon is responsible annually for over 700,000 deaths worldwide, and it has been estimated that this number will increase exponentially [1,2]. *E. coli*, *S. aureus*, *Enterococcus* spp., *P. aeruginosa*, *Kleibsiella* spp., coagulase-negative *Staphylococci*, and *Candida* spp. are among the most frequently isolated microbes in health care-associated infections [3]. The development of new antimicrobial treatments is a challenge that scientists are approaching through global collaboration. Unfortunately, developing a new drug is an extremely expensive and long process that may take up to 17 years to be completed, and as a consequence, the discovery of new antibacterial agents has dropped by more than 50% [4], while the development of bacteria resistance has been increasing and very rapidly spreading. A parallel approach is drug repositioning, meaning the identification of new clinical targets for a drug already tested, approved, and even marketed [5]. This approach could cut costs and speed up the approval timelines, as data on preclinical studies, pharmacokinetics, pharmacodynamics, and toxicity have already been reported. Drug repositioning has been a very promising approach to finding possible therapies against COVID-19, leading to the identification of several possible treatments, such as antivirals (i.e., Lopinavir/Ritonavis, Remdesivir), immunosuppressors (i.e., Ruxolitinib, Tocilizumab, Eculizumab), and immunomodulators (i.e., Camostat, Interferons, Sargramostin). Currently, in the USA, more than 1000 clinical trials employing one of these drugs or a combination of them have been undertaken, and additional trials are ongoing in the rest of the world [6,7,8].

Different strategies can be applied to drug repurposing. First, non-antimicrobial approved drugs can be tested directly on cell-based models to sort out those displaying antimicrobial activity. This strategy can be undertaken even in a high-throughput mode [9,10,11]. It is a phenotype-based process and therefore it does not clarify the action mechanism of the new active drugs identified [12]. Once a drug and its microbiological target are clarified, it is possible to perform a screening on barcode mutant libraries in order to identify the relative molecular target. Here, thanks to chemogenomic profiling that follows haploinsufficiency profiling or homozygous profiling assays, pathways, or even proteins whose activity were selectively modified by the presence of the drug, are identified. This provides important information on the biological effects of the tested molecule [13,14,15,16]. For example, following this approach, the molecular target of psychoactive drugs, such as drofenine, clozapine, propiomazine, and metergoline, were suggested to be small ribosomal subunit and cytochrome c oxidase (COX17), the terminal component of the mitochondrial respiratory chain [17]. Chemogenomic profiling is a very powerful tool that can be applied to a limited number of microbes, as mutant libraries were only developed for the main model organisms (i.e., yeasts *S. cerevisiae*, *C. albicans*, *S. pombe* and bacteria *E. coli* and *P. aeruginosa*). An alternative approach to identifying the antimicrobial activity of a drug is based on screening for a clear phenotype in more clinically relevant environments, for example, on whole-animal systems [18]. In this case, other tests (biochemical, genomic, and computational) are necessary to identify the molecular target(s) [12]. Screenings can also be performed in silico by taking advantage of recent improvements of high-performance computers, artificial intelligence (AI) [19], and the availability of a vast amount of biological, chemical, and pharmacological data.

There are almost 50 drugs that have been approved to be used on new targets due to drug repositioning initiatives [2,20,21,22]. Among those, amphotericin B, a known antifungal, is now approved to be used on visceral leishmaniasis, while chlorcyclizine, usually used to treat allergic reactions, can be employed as an antiviral, and the antibacterial doxycycline could be employed against malaria [23,24,25]. The first-generation antipsychotic haloperidol can induce ferroptosis in hepatocellular carcinoma cells. Ferroptosis is connected with the accumulation of Reactive Oxygen Species (ROS), and in this condition, haloperidol increases the cellular concentrations of GSH and Fe^2+^ and lipid peroxidation. [26,27,28]. Haloperidol is now employed in cancer preclinical and clinical studies. The antidepressants fluoxetine has been found to be beneficial against COVID-19 infections [6]. Taking fluoxetine lowers the risks of intubation and death by reducing the “cytokine storm” [29,30]. Moreover, several groups have reported the capacity of antidepressants and antipsychotics to inhibit the growth of different microbes, many of which are related to healthcare-associated infections. Overall, repurposed drugs could be used alone or, even better, in association with known antimicrobials to potentiate their actions. The latter strategy allows lowering the drugs’ concentration and diminishing their possible toxicity and, at the same time, maximizing their joint action and preventing antimicrobial resistance development [31]. This strategy was already proven successful [32,33,34]. These studies suggest that these molecules could be integrated into a drug repositioning pipeline. This review aims at listing all the available data, reporting the microbiological targets identified upon treatment and discussing these findings. More importantly, the novelty of this review is that it highlights some important implications that the use of antidepressants could have on the ecology of the human gut.

To identify all the relevant manuscripts for this perspective review, several bibliographic searches were undertaken from May to August 2021 on “PubMed”, “Elsevier’s Scopus”, Web of Science Clarivate Analytics, and “Google Scholar”. The searches included several different combinations of keywords (i.e., sertraline-antimicrobial, sertraline-repurposing, sertraline-fungi, SSRIs-bacteria, etc.).

## 2. Brief Overview of the Actions of Antidepressants and Antipsychotics

Depression has been mainly correlated with alterations in the noradrenergic and serotonergic functions. The link between a lower activity of the serotonin pathways and the development of depression is almost 50 years old. Indeed, in the 1950s, it was found that iproniazide and imipramine could improve the condition of depressed people and later, it was found that iproniazide, a MonoAmine Oxidase Inhibitor (MAOI), and imipramine, a Tricyclic Antidepressant (TCA), were able to inhibit the reuptake of monoamines and had the capacity to increase the effect of serotonin at the synapses [35]. Since the discovery of the first MAOI and TCA, many more variant molecules were developed. The lack of specificity of these drugs causes several undesirable side effects including toxic ones. To overcome these problems, Selective Serotonin Reuptake Inhibitors (SSRIs) were developed, a discovery that improved the quality of life of patients suffering from depression, as SSRIs are more tolerated and safer than MAOIs and TCAs [35,36,37].

People suffering from psychotic behaviors (i.e., schizophrenic, aggressive, anxious, agitated behaviors) are mostly cured with neuroleptics and benzodiazepines [27,38,39,40,41]. These drugs act by blocking the dopamine receptors. These drugs act by: (a) blocking dopamine receptors, or (b) combining the inhibition of serotonin uptake and dopamine receptors, and producing side effects on cholinergic, histaminic, and adrenergic receptors [42,43] or also (c) by promoting the activity of the neurotransmitter gamma-aminobutyric acid [44] (GABA).

## 3. Repurposing of Antidepressant and Antipsychotic Drugs

Recent reports have shown that antidepressants may have a positive effect on other pathologies. Indeed, besides their “classical” use, TCAs and SSRIs were beneficial when administered to persons suffering from irritable bowel syndrome. The dose of antidepressants used to treat patients with irritable bowel syndrome is lower than the one employed to treat psychiatric disorders. With this lower dose, no effect on anxiety or depression were generally reported [45,46,47]. Moreover, desipramine and fluoxetine reduce the risk of colitis in animal models, even though these data have to be confirmed in clinical trials [46]. The SSRIs fluoxetine, paroxetine, sertraline, escitalopram, and citalopram have been proven to reduce several premenstrual syndrome symptoms, although in some cases, side effects have also been reported with a dose-depended relationship [48,49]. Interestingly, sertraline had also positive effects in women suffering from premenstrual syndrome and recurrent vulvovaginal candidiasis. Indeed, in these patients during the cure, no recurrent episodes of yeast infection were reported [48,50]. This effect is not surprising, as many antidepressants have been found to have a negative effect on the growth of *Candida albicans* (see Table 1).

## 4. *Candida albicans*, a Common Target of Antidepressants and Antipsychotics

Many antidepressants and antipsychotics showed to be effective, at least in vitro, against several types of fungi such as *A. fumigatus*, *A. flavus*, *A. terreus*, and *C. neoformans*, and, especially, against *Candida* spp., particularly *C. albicans* (see Table 1 for details). *Candida albicans* is a member of a healthy microbiota, colonizing the gastrointestinal tract, skin, and oral cavity [79,80,81]. Unfortunately, alterations in the host immune response or its microbiota or changes in the environment can stimulate the growth of *C. albicans*, a phenomenon that can cause thrush, vaginal yeast infections, diaper rash, and even more serious infections especially in immunocompromised individuals [82]. Moreover, this yeast can easily colonize implanted medical devices thanks to its capacity to form stable biofilms, a contamination that could lead to bloodstream infections and even invasive systemic infections of organs or tissues [83]. Biofilm growth leads to inherent resistance to antimicrobial agents, a phenomenon connected with the increased expression of efflux pumps, the formation of the extracellular matrix, and the presence of persister cells [84,85]. Antifungal drugs used nowadays target ergosterol biosynthesis or 1,3-β-D-glucan synthesis. With the development of resistance and the fact that high doses of antimicrobials can cause liver and kidney damages [86], new molecules active against *C. albicans*, preventing biofilm formation or destroying its integrity, are certainly needed [87,88]. Therefore, the promising antimicrobial capacity of antidepressants and antipsychotics should be further investigated. In parallel, as this yeast is considered a model organism, the identification of the mechanism of action of a new antimicrobial is aided by the possibility to perform chemogenomics studies, as mutant libraries are available, as well as to perform DNA transformation, epitope tagging, and immunoprecipitation, to name a few.

## 5. Possible Antimicrobial Activities of Antidepressants and Antipsychotics

All three classes of antidepressants and many of the antipsychotics have antimicrobial activity (see Table 1); interestingly, most of the targeted microbes are isolated in healthcare-associated infections. Often, the reported biological effects following drug treatments are similar for many of the tested microbes. By looking at Table 1, it is clear that the consequences of the treatments have been described especially in *Candida* spp. In this yeast, antidepressants or antipsychotics often negatively influence key virulence factors, a phenomenon with negative consequences on the capacity of pathogenic fungi to attack the human body. Indeed, frequent drug targets are the formation of hyphae, which is one of the first steps in biofilm formation, the enzymes aspartyl proteinases, key virulence factors able to degrade many human proteins (i.e., mucin, immune system molecules, endothelial cell proteins, and coagulation factors), and phospholipase enzymes, proteins able to break the ester bonds of phospholipids, inducing cell lysis [89]. Active proteinase and phospholipase support the process of pathogenesis [90]. Many drugs have been shown to disrupt cell membrane’s integrity, sometimes also acting at the level of lipid rafts. The latter are areas of the membrane that are particularly rich in sphingolipids and are involved in membrane trafficking, control of Na^+^/K^+^ balance, and pH homeostasis [91]. Damage to the membranes can induce cell lysis or autophagy, the latter being a self-degrading process that can be also be directly induced by some antifungals [92]. Autophagy is correlated with mitochondria depolarization, another effect often observed when yeasts are treated with antidepressants and antipsychotics. The working concentrations reported in Table 1 are within the same range of those of the known antimicrobials and antibiotics (for *C. albicans*, the MIC_90_ of fluconazole is around 3 µM). Many works reported that these drugs work synergistically with azoles, especially fluconazole, and with amphotericin B. All of the combinations display a fractional inhibitory index (FICI or FIX) well below 0.5 [93]. This is a positive outcome, as these synergistic combinations could reduce the antibiotic concentrations used to treat an infection, in parallel reducing the toxic side effects and preventing the development of resistance [31].

Among the SSRIs, sertraline is the most studied, found to be active in some cases already at the concentration of 9 µM. This drug was tested on more than 20 microbes including yeast and bacteria. On yeast, and especially on *C. albicans*, it inhibits virulence factors such as hyphal formation and the secretion of aspartyl proteinases, kills yeast, and inhibits biofilm formation. Moreover, this drug works synergistically with fluconazole [50,51,58]. In bacteria, sertraline was shown to inhibit growth and to work synergistically with other antibiotics, but no further details have been reported [60,61,62,63]. To our knowledge, sertraline is also the only drug being employed as an antifungal in humans, specifically, against cryptococcal meningitis. Sertraline was initially demonstrated to be able to potentiate the action of azoles against cryptococcal microbes [60]; later, it was tested in a Phase II study [94] and a follow-up Phase III study in HIV patients infected by this fungus, but with no convincing results [95].

Fluoxetine, an SSRI, was found to be active against bacteria, fungi [50,56,64,67], and, interestingly, even a virus. Indeed, this drug was able to inhibit the viral RNA and proteins of *Coxsackievirus* [66]. The latter is an enterovirus, a small non-enveloped RNA virus, member of a genus responsible for different life-threatening conditions such as encephalitis or myocarditis [96]. Clorgyline, a MAOI, is active against *C. albicans* and *C. glabrata* where it inhibits efflux pumps and positively interacts with fluconazole [56,70]. A second MAOI, phenelzine, was found instead to be active against *Salmonella* [71]. Works based on TCAs showed that this class of molecules was active against *Candida* spp., where they inhibited hyphal formation, lysed the cells, and killed them even in a mature biofilm [72,73]. Moreover, the TCA amoxapine is active against *Salmonella* and *Y. pestis* [54,71]. Interestingly, some TCAs and the antipsychotic thioridazine are capable to resensitize methicillin-resistant *S. aureus* (MRSA) to β-lactam [97,98,99]. This is relevant, as it opens the possibility to use this drug, alone or in combination with known antibiotics, to treat resistant bacteria.

The neuroleptics aripiprazole and bromperidol can inhibit biofilm formation by *Candida* spp., but the second was found to work synergistically with spectinomycin to inhibit the growth of *Mycobacterium smegmatis* and *M. tuberculosis* [55,75]. Benzodiazepines are active against *C. albicans* and can inhibit biofilm formation; they are also active against *S. aureus*, *E. faecalis*, *E. coli*, *P. aeruginosa,* and *A. baumanii* [10,76,77,78].

## 6. Possible Mechanisms of Action

Antidepressants and antipsychotics display antimicrobial activity on a wide range of microbes, as reported in Table 1. However, the examined reports do not include specific details on their mechanism of action and molecular targets. The following paragraphs summarize what is known so far on this topic.

### 6.1. Inhibition of Efflux Pumps

**Figure 1:**
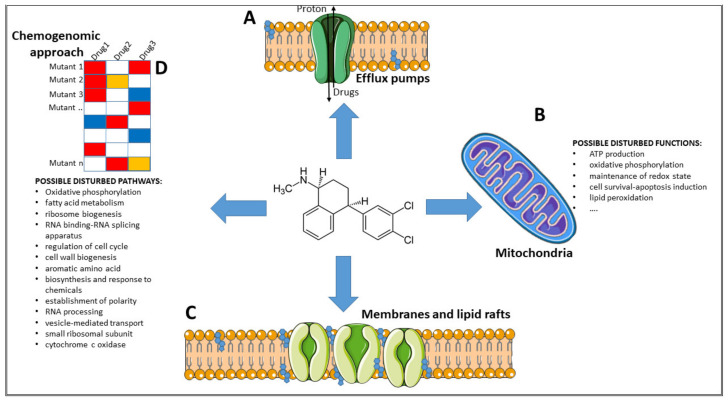
Proposed mechanisms of action. Few are the mechanisms of action suggested for antidepressants and antipsychotics. They involve inhibition of efflux pumps (**A**), disturbance of mitochondria (**B**) (a list of possible functions that can be affected in the organelle are reported), and disruption of the integrity of membranes or lipid rafts (**C**). Moreover, with chemogenomics and the use of barcoded mutants (**D**), pathways and molecular targets of different psychoactive drugs were discovered in the model organisms *S. cerevisiae* and *C. albicans*. Future studies will need to confirm these findings. Part of the graph was made using https://smart.servier.com/ (accessed on 1 July 2021).

A direct target of SSRIs, and partially of some TCAs, is the human Serotonin Transporter, a membrane-bound Na^+^-dependent solute carrier with 12 putative transmembrane domains. Its action allows the import of serotonin inside cells, and antidepressants inhibit this action. It has been suggested that, in microbes, the same molecules lower the activity of other proteins transporters such as xenobiotic efflux pumps (Figure 1). Indeed, overexpression of the efflux pumps Cdr1, Cdr2, or Mdr1 is a common way adopted by *C. albicans* to detoxify itself from xenobiotics [100]; *P. aeruginosa* does the same with MexAB-OprM and MexXY-OprM [101], and *S. enterica serovar Typhimurium* increases the levels of AcrB to excrete β-lactam antibiotics [102]. Inhibiting the removal of a toxic compound, such as an antibiotic, would therefore allow the latter to exert its action within cells. This idea is supported by the observation that the activity of *C. albicans* transporters overexpressed in multi-drug-resistant strains and expressed in *S. cerevisiae,* can be inhibited by the antidepressants clorgyline [70].

### 6.2. Inhibition of Mitochondria Activity

As reported in several works, the action of antidepressants and neuroleptics in microbes involves different biological processes, and it is suggested that several different components could be the target of these drugs [61]. Indeed, treatment of a *C. albicans* mutants library with nortriptyline identified mutants that are specifically sensitive or tolerant to this drug and others that share the same phenotype when treated with common antifungals [73]. The work suggests that mitochondria are one of the targets of nortriptyline (Figure 1), although it is not clear which function, carried out by this organelle (i.e., ATP production and oxidative phosphorylation, maintenance of the redox state, cell survival or apoptosis induction, lipid peroxidation), is affected. Inhibition of mitochondrial activity is displayed also when yeasts are treated with other central nervous systems drugs [103].

### 6.3. Interference with Membrane Integrity

It has been suggested that neuroleptics such as aripiprazole can interfere with Lanosterol 14 alpha-demethylase or CYP51, similarly to azoles, disrupting lipid rafts and inducing membrane damage [74]. The antipsychotic phenothiazine and the antidepressant nortriptyline have also been reported to disrupt cell membranes [72,104,105]. As mentioned earlier, TCAs and thioridazine, an antipsychotic, are able to reverse the resistance of MRSA to β-lactam. It has been reported that do so, TCAs act by repressing the expression of β-lactam resistance genes, while thioridazine acts by inhibiting the expression of genes involved in cell wall biosynthesis [98,99].

### 6.4. Possible Disturbed Pathways

**Figure 2:**
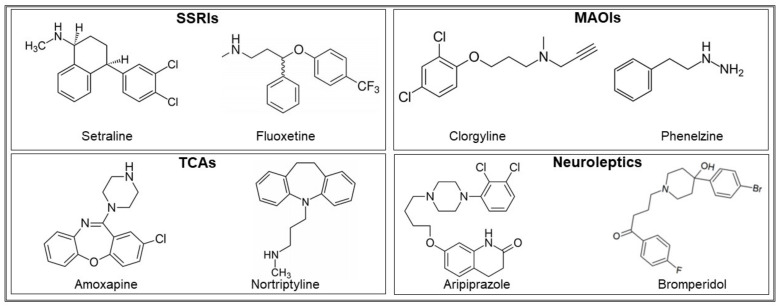
Chemical structure of some SSRIs, MAOIs, TCAs, and neuroleptics which highlights similarities and differences between the different chemicals.

Chemogenomic profiling with a haploinsufficiency approach focused on *C. albicans* mutants identified the following processes and systems as possible targets of nortriptyline: oxidative phosphorylation, fatty acid metabolism, ribosome biogenesis and machinery, RNA binding, and the RNA splicing apparatus [73,106]. Following the same approach, the targets of the TCA chlorpromazine in *S. cerevisiae* were suggested to be the regulation of cell cycle, cell wall biogenesis, aromatic amino acid biosynthesis, and response to chemicals [106]. With the same approach, Ericson et al., showed that in *S. cerevisiae*, the most notable effect of fluoxetine was on the establishment of polarity, that of paroxetine was RNA processing, and that of sertraline was vesicle-mediated transport [107]. Hillenmeyer et al. reported that, in the same microbe, the small ribosomal subunit and cytochrome c oxidase are the targets of psychoactive drugs [17]. The heterocyclic compounds acridines have been widely used also as antibacterial agents, due to their capacity to bind DNA and interfere with the activity of the enzymes topoisomerase I and II [108]. It has been suggested that two or more benzene rings, a common feature of acridine and of some antidepressants and antipsychotics (see Figure 2), could be among the key functional groups for antimicrobial activity [53].

## 7. Implications of the Antimicrobial Activity of Antidepressants in Human Gut Ecology

In the previous sections, we described how antidepressants and antipsychotics negatively influence the growth and physiology of different microbes, both bacteria and fungi. This observation has broader therapeutic implications, as many microbes are symbionts or opportunistic microorganisms in the human body, including skin and gut.

Recently, phenelzine, desipramine, venlafaxine, bupropion, aripiprazole, and (S)-citolapram have been tested for this property also in isolated commensal bacteria. The results have shown that these drugs can inhibit the growth of phyla that are dominant within the human microbiota such as *Bifidobacterium animalis* and *Bacteroides fragilis* [109]. This work strongly suggests the capacity of antidepressants to act as antimicrobials also on beneficial microbes residing in the human gut.

It is known that the gut forms a two-direction communication system with the brain, forming the so-called “gut–brain axis”. Serotonin is a neurotransmitter whose concentration is fine-tuned in our body. Indeed, low levels of this neurotransmitter have been correlated with the development of depression, while high levels have been linked also to colitis [110,111]. The gut microbiota was implicated in regulating the levels of serotonin [110], and some of the microbes residing in the gut can produce serotonin (i.e., *Lactococcus lactis subsp. cremoris*, *L. lactis subsp. Lactis*, *Lactobacillus plantarum*, *Streptococcus thermophilus*, *E. coli* K-12, *Morganella morganii*, *Klebsiella pneumonia*) [110,112,113]. So far, the possibility that modifications of the gut microbiota could negatively influence serotonergic signaling has only been suggested in pre-clinical studies, as data from clinical trials are still very limited [110].

Moreover, it was reported that people suffering from depression frequently displayed an altered gut microbiota, with a decreased richness and diversity of species [114]. For example, Lin et al. showed that depressed patients display a different gut composition with more bacteria of the phylum *Firmicutes*, less *Bacteroidetes*, and more bacteria of the genera *Prevotella*, *Klebsiella*, and *Streptococcus* [115], while Naseribafrouei et al. showed that *Bacteroidales* are increased in depressed patients who have a decreased level of the *Lachnospiraceae* family (a more exhaustive list can be found in a recent review [116]). It was reported that the frequent use of antibiotics was correlated with an increased risk of developing depression or anxiety [65].

Probiotics have been recently tested to re-establish a healthy microbial status, due to their ability to balance the dysbiosis created under the depressive state [117]. As an example, the psychobiotic *B. breve* A-1 improved depressive symptoms in patients with schizophrenia [118], while a mixture of *L. acidophilus*, *L. casei*, and *Bifidobacterium bifidum* was beneficial on depressive symptoms [119]. At the moment, many psychobiotics, being them prebiotics, probiotics, postbiotics, and even single molecules involved in the gut microbial signaling, are being tested to identify any possible therapeutic benefits [116,120].

Previous reports showed that patients suffering from depression display microbial dysbiosis. As many of these studies were conducted in patients with depression, who were often being treated for it at the time of the analyses, more studies are necessary to describe the gut microbiome before and after medical treatments, so to understand if and how antidepressants could influence dysbiosis. Moreover, taking antidepressants could affect specifically those microbial strains able to produce serotonin, possibly adding a negative factor to the overall compromised health system, which is an extra factor to considered. A recent hypothesis suggests that shifts in the gut microbial composition upregulate pro-inflammatory pathways leading to the worsening of depressive symptoms, a theory that implicates also the immune system in this complex balance [116].

## 8. Conclusions

The development of antibiotic resistance is a global threat. To try to solve this problem, new antibiotics or antimicrobials should be developed, a task that has to face many different challenges. As a complement to new drug discovery, drug repositioning could be investigated. In this review, the antimicrobial capacity of antidepressants and antipsychotics identified by different laboratories has been summarized. Many of these drugs have been found to disturb the integrity of the cell membrane, mitochondria activity, and critical *Candida* spp. virulence factors (i.e., hyphae formation, SAP, and phospholipase enzymes). Less is known on their effects on bacteria. The premises are encouraging; however, more aspects need to be investigated in depth before some of these drugs could be labelled as true antimicrobials. To reach this point, it will be necessary to:

- Clearly identify the microbial molecular target(s) of each potential new drug, especially in bacteria
- Describe the connection between the administration of these drugs and their influence on the gut microbiome, after a short and/or prolonged administration, and in the frame of the gut–brain axis
- Pinpoint the concentration at which these molecules would be active as antimicrobials, a parameter that should be equal to, or even lower than, the one used to treat the original disease
- Identify new uses, such as investigate the possibility of topical application
- Test whether, when combined with other known antimicrobials, they potentiate the overall effects of the latter and at the same time, allow lowering antimicrobials’ global concentration and their possible correlated toxic effect
- Better understand which of these drugs may have an acridine-like effect on DNA and which are more active at the level of mitochondrial functions.

## Figures and Tables

**Table 1:** List of different antidepressants and antipsychotics; their “classical” action in humans is correlated to their described antimicrobial activity. For each drug. the microbial target, the effective concentration at which antimicrobial activity is observed, and the physiological effects on the listed microbes are presented. Almost all papers report in vitro testing, with the exception of [50,51,52,53,54,55], which report some in vivo testing.

Drug	Microbial Target	Concentration Effective on Microbes	Physiological Effects on Microbes	References
ANTIDEPRESSANTs—SSRIs				
Sertraline (third generation of selective serotonin reuptake inhibitors)	*C. albicans*, *C. glabrata*, *C. tropicalis*, *C. parapsilosis*, *C. dubliniensis*, *C. krusei*, *A. fumigatus*, *A. flavus*, *A. terreus*, *C. neofromans*, S. cerevisiae, *A. baumanii*, *H. influenzae*, *C. jejuni*, *H. pylori*, *S. aureus*, *P. aeruginosa*, *S. epidermidis*, *E. faecalis*, *C. difficile*, *B. fragilis*, *Prevotella* spp	9–775 µM (3–237 µg/mL)	Fungicidal; inhibits hyphal elongation and phospholipase activity, reduces secreted aspartyl proteinases (SAP) production, inhibits fungal viability, has antifungal and anti-biofilm effects, displays a synergistic effect with fluconazole, causes mitochondrial depolarization and cell membrane damage, induces autophagy	[50,51,56,57,58,59,60,61,62,63]
Fluoxetine (third generation of selective serotonin reuptake inhibitors)	*C. albicans*, *E. coli* *P. aeruginosa*, *S. aureus*, *Coxsackievirus*, *E. coli*, *A. baumanii*	130 µM–13 mM (40–4000 µg/mL)	Inhibits cell growth; promotes mitochondrial depolarization and membrane damage; decreases metabolic activity of mature biofilms; displays synergistic interaction with azoles such as fluconazole, downregulates SAP genes expression and extracellular phospholipase activity, inhibits bacterial growth and synergizes with antibiotics, reduces the synthesis of viral RNA and proteins.	[52,56,58,64,65,66,67]
Paroxetine (third generation of selective serotonin reuptake inhibitors)	*C. albicans*, *E. coli*, *A. baumanii*	110–282 µM (40–101 µg/mL)	Inhibits cell growth; promotes mitochondrial depolarization and membrane damage in yeast, inhibits bacterial growth.	[56,58,67,68,69]
ANTIDEPRESSANTs—MAOIs				
Clorgyline (MonoAmine Oxidase Inhibitor)	*C. albicans*, *C. glabrata*	5.1 µM (1.4 µg/mL)	Broad-spectrum inhibitor of several fungal efflux pumps, displays a synergistic interaction with fluconazole	[56,70]
Phenelzine (MonoAmine Oxidase Inhibitor)	*Salmonella*	30–100 µM (4–14 µg/mL)	Inhibits TYR oxidoreductase	[71]
ANTIDEPRESSANTS—TCAs				
Doxepin (increases the levels of norepinephrine, along with blocking histamine, acetylcholine, and serotonin)	*C. albicans, C. glabrata, C. parapsilosis, C. krusei, C. utilis*	716 µM (200 μg/mL)	Inhibits hyphae and biofilm formation, kills cells in a mature yeast biofilm	[72]
Imipramine (increases the levels of serotonin and norepinephrine and blocks some serotonin, adrenergic, histamine, and cholinergic receptors)	*C. albicans, C. glabrata* *C. parapsilosis, C. krusei, C. utilis*	142 µM (40 μg/mL)	Inhibits hyphae and biofilm formation, kills cells in a mature yeast biofilm	[72]
Nortryptiline (blocks the reuptake of norepinephrine, binds to alpha-adrenergic, histaminergic, and cholinergic receptors)	*C. albicans, C. glabrata, C. parapsilosis, C. krusei, C. utilis*	190 µM (50 μg/mL)	Inhibits hyphae and biofilm formation, kills cells in a mature biofilm, induces cell lysis, and displays synergistic activity with amphotericin B	[72,73]
Amitriptyline (Inhibits the reuptake of serotonin and norepinephrine)	*Staphylococcus* spp., *Bacillus* spp., *V. cholerae*, *Micrococcus* spp, *L. sporogenes*, *Citrobacter* spp.	36–722 µM (10–200 μg/mL)	Inhibits microbial growth	[53]
Amoxapine (Reduces the uptake of serotonin and noradrenaline)	*Salmonella*, *Y. pestis*	1–100 µM (0.3–30 µg/mL)	Inhibits GUS-mediated hydrolysis of d-glucuronides, reduces cytotoxicity in murine macrophages	[54,71]
ANTIPSYCHOTIC—NEUROLEPTIC				
Aripiprazole (partial agonist of serotonin and dopamine receptors)	*C. albicans*	11–111 µM (5–50 µg/mL)	At low doses, it inhibits biofilm formation, as well as yeast-to-hyphal transition and flocculation; at high doses, disrupts lipid rafts, induces membrane damage	[74]
Bromperidol (antagonist of the dopamine receptor)	*Mycobacterium smegmatis*, *M. tubercolosis*, *C. albicans*, *C. glabrata*, *A. terreus*	119–142 µM (50–60 µg/mL)	Acts synergistically with spectinomycin on *Mycobacterium*, is bactericidal, interacts positively with azoles	[55,75]
ANTIPSYCHOTIC—BENZODIAZEPINE				
Diazepam (increases the effect of the neurotransmitter GABA)	*C. albicans*	108 µM–14 mM (31.25–4000 μg/mL)	Inhibits growth, hyphae formation, and biofilm growth	[76]
Lorazepam (enhancer of the effect of the inhibitory neurotransmitter gamma-aminobutyric acid on GABA receptors)	*C. albicans*	96 µM–12 mM (31.25–4000 μg/mL)	Inhibits growth, hyphae formation, and biofilm growth	[76]
Midazolam (promotes the action of GABA)	*C. albicans*, *S. aureus*, *E. faecalis*, *E. coli*, *P. aeruginosa*, *A. baumanii*	95 µM–12 mM (31.25–4000 μg/mL)	Inhibits growth, hyphae formation, and biofilm growth, inhibits bacterial growth	[76,77,78]
ANTIPSYCHOTIC—ATYPICAL				
Zotepine (High affinity to dopamine receptors, affects serotonin receptors, its active metabolite, norzotepine, serves as a potent norepinephrine reuptake inhibitor)	*C. albicans*	0.1–40 µM (0.03–12 µg/mL)	Inhibits biofilm development	[10]

## Data Availability

Data sharing not applicable.
